# The Potential for Decision Support Tools to Improve the Management of Root-Feeding Fly Pests of Vegetables in Western Europe

**DOI:** 10.3390/insects11060369

**Published:** 2020-06-13

**Authors:** Rosemary Collier, Dominique Mazzi, Annette Folkedal Schjøll, Quentin Schorpp, Gunda Thöming, Tor J. Johansen, Richard Meadow, Nicolai V. Meyling, Anne-Marie Cortesero, Ute Vogler, Michael T. Gaffney, Martin Hommes

**Affiliations:** 1 Warwick Crop Centre, School of Life Sciences, University of Warwick, Wellesbourne, Warwick CV35 9EF, UK; 2 Agroscope, Research Division Plant Protection, Müller-Thurgau-Strasse 29, 8820 Wädenswil, Switzerland; dominique.mazzi@agroscope.admin.ch; 3 Norwegian Institute of Bioeconomy Research (NIBIO), Division of Biotechnology and Plant Health, P.O. Box 115, NO-1431 Ås, Norway; Annette.Folkedal.Schjoll@nibio.no (A.F.S.); Gunda.Thoeming@nibio.no (G.T.); Tor.Johansen@nibio.no (T.J.J.); 4 Julius Kühn Institute (JKI), Institute for Plant Protection in Horticulture and Forests, Messeweg 11-12, D-38104 Braunschweig, Germany; quentin.schorpp@julius-kuehn.de (Q.S.); ute.vogler@julius-kuehn.de (U.V.); martin.hommes@julius-kuehn.de (M.H.); 5 Department of Plant Sciences, Norwegian University of Life Sciences, P.O. Box 5003, N-1432 Ås, Norway; richard.meadow@nmbu.no; 6 Department of Plant and Environmental Sciences, University of Copenhagen, Thorvaldsensvej 40, 1871 Frederiksberg C, Denmark; nvm@plen.ku.dk; 7 IGEPP, INRAE, Institut Agro, Univ Rennes, 35000 Rennes, France; anne-marie.cortesero@univ-rennes1.fr; 8 Horticultural Development Department, Teagasc, Ashtown, D15DY05 Dublin 15, Ireland; Michael.Gaffney@teagasc.ie

**Keywords:** fly larvae, *Delia radicum*, *Delia floralis*, *Chamaepsila rosae*, Delia platura, Delia florilega, Delia antiqua, monitoring, forecasting, decision support, integrated pest management

## Abstract

Several important vegetable crops grown outdoors in temperate climates in Europe can be damaged by the root-feeding larvae of Diptera (*Delia radicum*, *Delia floralis*, *Chamaepsila rosae*, *Delia platura*, *Delia florilega*, *Delia antiqua*). Knowledge of pest insect phenology is a key component of any Integrated Pest Management (IPM) strategy, and this review considers the methods used to monitor and forecast the occurrence of root-feeding flies as a basis for decision-making by growers and the ways that such information can be applied. It has highlighted some current management approaches where such information is very useful for decision support, for example, the management of *C. rosae* with insecticidal sprays and the management of all of these pests using crop covers. There are other approaches, particularly those that need to be applied at sowing or transplanting, where knowledge of pest phenology and abundance is less necessary. Going forward, it is likely that the number of insecticidal control options available to European vegetable growers will diminish and they will need to move from a strategy which often involves using a single ‘silver bullet’ to a combination of approaches/tools with partial effects (applied within an IPM framework). For the less-effective, combined methods, accurate information about pest phenology and abundance and reliable decision support are likely to be extremely important.

## 1. Introduction

Several important vegetable crops grown outdoors in temperate climates can be damaged by the root-feeding larvae of Diptera, specifically the larvae of *Delia radicum* (L.) (cabbage root fly), *D. platura* (Meigen) (bean seed fly), *D. florilega* (Zetterstedt) (root fly), *D. antiqua* (Meigen) (onion fly), *D. floralis* (Fallén) (turnip fly) and *Chamaepsila rosae* (Fabricius) (carrot fly). All are well-adapted to temperate climates and have effective ways of surviving the winter, mainly, but not exclusively, through pupal diapause. These species infest a wide range of important crops, being pests of Brassicaceae, e.g., cabbage, cauliflower (*D. radicum, D. floralis, D. platura*), Apiaceae e.g., carrot, parsnip (*C. rosae*), Alliaceae, e.g., onion (*D. antiqua, D. platura, D. florilega)* and several other plant families, e.g., pea, bean, sweetcorn, courgette (*D. florilega*, *D. platura*).

Whilst all species damage the root systems of host plants (and some occasionally attack above-ground plant parts as well), the economic significance of the damage depends on whether it reduces plant growth and, in terms of produce quality, whether it affects the marketable part of the plant. A small amount of root damage may have only a minor impact on the growth or quality of leafy or flowering brassicas like broccoli and cauliflower but have a considerable impact on the quality of root crops such as carrot, radish, turnip or swede (rutabaga). Root-feeding fly larvae themselves are difficult targets for insecticidal control because they are in the soil or inside the plant tissue, and non-systemic insecticides applied as sprays do not readily penetrate to reach the target. Thus, seed treatments, granule treatments and drench treatments are likely to be more effective than foliar sprays. Adult flies are also difficult targets because there need to be opportunities for them to acquire a sufficient dose of insecticide, either through direct contact or through contact with treated surfaces, and they are sometimes only active within the crop for part of the day.

Due to environmental and human health considerations, many effective insecticidal active ingredients have been banned in several European countries, making crop protection more challenging for the growers. Some products have been ‘lost’ as a result of new legislation, and, for specialty crops, such as vegetables and salads, their relatively small market does not justify/offset the costs of renewing registrations lost as a result of updated legislation. A range of alternative approaches to insecticides are available as components of Integrated Pest Management (IPM) strategies. IPM approaches include host plant resistance [1], sterile insect release [2], entomopathogenic nematodes [3], entomopathogenic fungi [4,5,6] and predatory mites [7], physical barriers [8,9,10,11], cultural methods like crop rotation, companion planting, intercropping and undersowing [12,13], trap crops [14,15], the application of volatile organic compounds [16,17] and the development of a management system using the ‘push–pull’ approach [18]. There is obviously scope for refining and combining some of these approaches and for evaluating them in different locations and cropping systems. However, with the exception of physical barriers, few of these have been implemented commercially to date and control still relies mainly on the use of chemical insecticides in many countries.

Knowledge of pest insect phenology is a key component of any IPM strategy. To quote Barzman et al. (2015): ‘In an ideal world, all farmers would monitor pest populations and use forecasting systems prior to making a decision regarding control’ [19]. This review considers the approaches taken to monitor and forecast the occurrence of root-feeding flies as a basis for decision-making by growers and how they might be used now, and in the future, to improve the management of the root-feeding fly pests of vegetable crops. We also need to know how these approaches can provide added value to IPM strategies, and how the approaches are to be further developed and improved in order to continue to do so in the future. Monitoring and forecasting are used to determine the timing of crucial events in pest lifecycles so that management actions can be applied when they are likely to be most effective; the main aim being to reduce the negative impacts of unnecessary treatments [20] and to optimise the timing of control measures.

This paper has arisen from a European ERA-NET C-IPM project called FlyIPM (Integrated control of root-feeding fly larvae infesting vegetable crops), which considers all of these species. The project consortium consisted of nine partner organisations in eight European countries.

## 2. Approaches Used to Monitor Pest Infestations

The basic phenology of the root fly species considered here is relatively well understood and developmental biotypes of *D. radicum* and *D. floralis*, which complete diapause at different times in the year, have been identified [21,22]. Monitoring systems have been developed for all species, some of which have ‘discrete’ generations in many locations, although those of *D. platura*/*D. florilega* appear to merge, possibly because of the relatively rapid lifecycle and broader host range of this species. In addition, in areas where both early-emerging and late-emerging *D. radicum* populations are present [21], overlapping generations of these two biotypes may lead to continual ‘fly pressure’. The same situation of continuous ‘fly pressure’ may arise with overlapping emergence of *D. radicum* and *D. floralis*. *Delia floralis* is a significant pest only in the more northerly parts of Europe, e.g., Norway and Scotland. The monitoring approaches used for each species are summarized in Table 1.

Adult *D. radicum* can be captured in water traps [23] or on sticky traps, although it can be more difficult to identify them on sticky traps. Monitoring systems depend mainly on the flies’ attraction to particular wavelengths of light (yellow traps are usually employed) [24], although some also exploit the flies’ responsiveness to host plant volatiles. It is also possible to monitor changes in the numbers of eggs laid around selected host plants either by direct sampling of the substrate (soil or sand) [25] or by using ‘egg traps’ [26]. Since *D. floralis* infests the same host plants as *D. radicum*, monitoring methods are broadly similar.

Two studies indicated that blue and white traps are more effective than yellow traps [27,28] for capturing *D. platura*. More recently, a synthetic lure has been developed which can be used in conjunction with these traps [29]. Most countries do not monitor numbers of *D. platura* and *D. florilega* routinely, although routine monitoring is undertaken at one site (Wellesbourne, Warwickshire) in the UK using yellow water traps.

Adult *D. antiqua* can be captured in water traps or on sticky traps [30,31,32]. Research has been undertaken to determine the most effective colour for these traps (blue or white are effective) [32,33]. *Delia antiqua* is not monitored routinely in any northern European country, although in Norway there is a demand for such monitoring [34].

**Figure 1:**
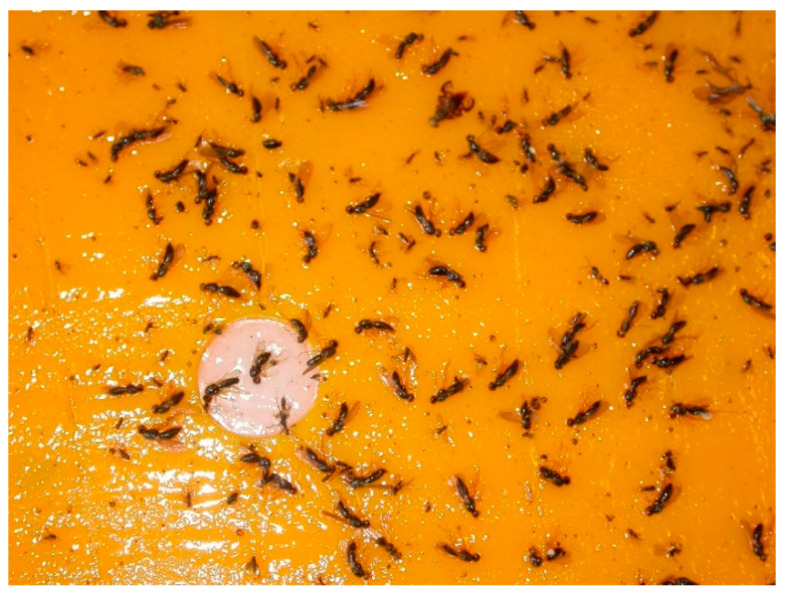
Adult *Chamaepsila rosae* on Rebell^®^ (Andermatt Biocontrol AG, Grossdietwil, Switzerland) sticky trap (image provided by Rosemary Collier, University of Warwick, Warwick, UK).

Adult *C. rosae* can be captured in water traps or on sticky traps but sticky traps are used in most instances (Figure 1). Again, trap colour (usually orange) is important [35]. Finch and Collier [36] also showed that inclining traps at 45° to the vertical made them more attractive to, and selective for, *C. rosae*, which prefers to land on the lower surface of the trap.

Most of the information gathered and disseminated about these species relates to their phenology (timing) rather than abundance. One exception is the use of information on numbers of *C. rosae* to deploy treatment thresholds. The use of thresholds for *C. rosae* in northern Europe was reviewed in 2009 following a European workshop on management of this species [37]. Treatment thresholds based on egg counts are used for the *D. radicum*/*D. floralis* complex in Norway [38]. In Germany, thresholds have been investigated for *D. radicum* [39], but are not in use any more due to changes and reductions in the availability of insecticides. The thresholds are only valid for head cabbage, broccoli and cauliflower and are related to the development of the crop. The availability of thresholds for each species within Western Europe is summarized in Table 1.

## 3. Approaches Used to Forecast Pest Infestations

The forecasting approaches used for each species are summarized in Table 1. Forecasting systems have been developed for five species: *D. radicum*, *D. floralis*, *D. platura*, *C. rosae* and *D. antiqua*. All but one of the systems forecast phenology rather than abundance; a Norwegian forecast for *D. floralis* is based on a damage threshold. Degree-day forecasts have been developed in North America for *D. radicum* [40,41,42], *D. platura* [43,44], and *D. antiqua* [45] and are presented on several advisory web sites in North America [46] and are obviously available for use in Europe. In the UK, simulation models have been developed for *D. radicum* and *C. rosae* [47], and in Germany there are simulation models for these species together with a preliminary model for *D. antiqua* [48]. In Norway, a degree-day model for *D. radicum* has been developed based on spring emergence and the oviposition period [34,49,50]. A comparable forecasting model based on degree-days for *D. radicum* is also available in Denmark based on local soil temperatures for individual postal code areas. All models require current weather data. The degree-day models use air temperature records. Air and soil temperatures are used in the UK, Norwegian and German simulation models. There has, to our knowledge, been no European-wide approach to developing forecasting tools for these pests; this might be a valuable approach in the future. The models or their outputs are disseminated in several ways. For example, in Norway, VIPS (Varsling Innen PlanteSkadegjørere) is an online open-source forecast and information service for decision support in integrated management of pests, diseases and weeds [51]. The current local emergence of *D. radicum* in Denmark is communicated online via the advisory service HortAdvice [52] for growers and advisors to observe when risks of oviposition by first- and second-generation flies are highest. In Britain, the Agriculture and Horticulture Development Board (AHDB) Pest Bulletin [53] is an on-line service with forecast outputs and some monitoring data. In Switzerland, information is provided via the “Gemüsebau Info”, an electronic bulletin coordinated by the competence centre for agricultural research, Agroscope, covering all growing regions and the three official languages. In Germany, ISIP (Information System for Integrated Plant Production) [54] provides advisors with relevant information on request. In France, information is given through the “Bulletins de Santé du Végétal (BSV)” coordinated by the regional agricultural chambers and available online [55].

## 4. Use of Decision Support

### 4.1. Insecticides

Overall, there are relatively few, or often no, effective insecticidal methods available for managing root flies. Those available are mostly applied prophylactically, irrespective of pest phenology and abundance. Examples are the use of module drench treatments for transplanted brassicas (e.g., use of chlorpyrifos, spinosad, and cyantraniliprole in some countries) and of seed treatments (e.g., tefluthrin) for certain crops. However, the number of seed treatments available has diminished in recent years, partly as a result of the banning of relevant insecticides on outdoor crops in the European Union, with neonicotinoids being a recent example [56].

Information on pest phenology and, in some cases, pest abundance is more useful for treatments applied as foliar sprays. This relates, for example, to control of *C. rosae*, particularly the second generation in most locations. The information is particularly useful when pyrethroids are used to control *C. rosae* with sprays; since these contact insecticides do not kill fly larvae, the timing of application is crucial to prevent egg laying [57]. The newer diamide insecticides, which have some systemic activity and have approval in some European countries, appear to have activity against both *C. rosae* larvae and adults, so timing may not be quite so critical [58]. If the prophylactic seed treatments that have been withdrawn are replaced by other treatments that require accurate timing, either of insecticides or biopesticides, or of arthropod natural enemies, then monitoring and forecasting systems will become increasingly important to time the application of such treatments.

As an example, in Norway, a Finnish temperature-based model for degree-day accumulation [59] is used in the VIPS system for forecasting the flight activity period for the first generation of carrot flies. In addition, growers are recommended to use yellow sticky traps to monitor activity. The growers are recommended to spray when the first flies occur but, based on field history and due to the lack of effective insecticides, some growers tend to wait a bit longer before applying the first insecticide spray. After the first insecticide application has been applied, growers are advised to spray when the trap catches four or more flies per trap per week [60]. Damage thresholds are also being used for the *D. radicum/D. floralis* complex in Norway, even though most producers use crop covers. The damage thresholds are related to the plants’ developmental stage and indicate the number of eggs per plant that can be tolerated before a reduction in growth and yield is expected. There is one threshold for newly planted cabbage (i.e., head cabbage, broccoli and cauliflower), and another threshold for cabbage that has been in the field for more than 4 weeks. The VIPS system produces warnings based on weekly observations of oviposition related to damage thresholds for the different development stages.

### 4.2. Physical Control

At present, the non-insecticidal control method used most widely is the deployment of physical barriers (mainly woven mesh covers) on certain crops (particularly root brassicas such as swede, turnip and radish, but also cabbage) and also on carrot crops grown in organic systems. In many districts of Norway, the application of crop covers to brassicaceous and apiaceous crops is the predominant control method, for conventional and organic growers alike.

In most cases, the use of covers has led to some changes in management practices [11]. Choice of mesh size can be critical, as more than one species of pest may need to be taken into account. For example, if other pests, such as flea beetles, aphids or diamondback moths, are able to access covered brassica crops, damage may be exacerbated due to the exclusion of natural enemies.

Information on pest phenology is useful with regard to the application and removal of physical barriers, to ensure that they are applied in a timely fashion and not removed until the risk of egg-laying has passed. It can be important to remove covers as soon as is feasible to avoid some of their adverse effects, often related to loss of light or an increase in temperature and humidity, which may include exacerbation of problems due to weeds, pathogens or other pests and the impairment of crop growth. It is also important to ensure that pests are not ‘trapped’ beneath crop covers as this can increase, rather than reduce, pest damage. Sometimes there is a need to remove the covers for a short period to weed the crop, and trials in Norway indicated that the morning is the best time of day in terms of avoiding colonisation by *D. radicum*/*D. floralis* [61]; similarly, female carrot flies are most active in the late afternoon [57]. A more detailed understanding of specific pest phenology may be required if crops are to be grown and covers to be applied more than once in the same field, for example for the cultivation of radish in the UK [11].

With the demise of certain insecticidal methods of control, crop covers may be used more widely, for example, to manage *D. platura/D. florilega* on legumes such as peas. In the UK, there appears to be one particularly large early peak in numbers, and discussions with growers and advisors have indicated that knowledge on how to forecast this peak would be very useful [62].

Exclusion fences are vertical barriers of insect netting that enclose the crop. Exclusion fences may be used without or with an insecticide incorporated in the threads. At least two of the root fly species (*C. rosae* and *D. radicum*) spend much of their time as adults in the field edges [63,64]. The females fly into the field to lay their eggs in the soil near the host plant and then fly back to the field edges. Due to this behaviour, vertical barriers have been shown to be effective in protecting the crop from root fly attack [8,9,10]. In certain areas of Norway, Fence^®^ (Vestergaard S.A., Lausanne, Switzerland) (i.e., exclusion fences with deltamethrin incorporated in the yellow threads) is widely used to protect brassica crops (especially swede) from infestation by *D. floralis* and *D. radicum*. However, since the fences must be in place before the adults come into the field, and as there is no need for timely removal of the fences, this control method derives little benefit from decision support [65]. Tests in Germany showed only small effects from use of vertical barriers [10], and this approach has, therefore, not been used commercially.

### 4.3. Avoidance through the Adjustment of Planting or Harvesting Dates

Some of the susceptible crops are sown/planted at regular intervals over a period of weeks or months in order to provide continuity of supply. In this case, it may not be possible to avoid periods when the risk of pest infestation is greatest. However, for certain crops, information about pest phenology can be used to delay/avoid planting or sowing during periods when the risk is particularly high [66,67]. One key example is delaying the sowing of carrot crops to avoid infestation by first-generation *C. rosae*. It has been shown (summarized by Collier and Finch [57]) that this can reduce considerably the numbers of flies that might infest the crop at the time of the second generation. This is because *C. rosae* does not disperse far compared with some other pest species, so the greatest risk of infestation by second-generation *C. rosae* is from the immediate vicinity. Similar results were obtained from a research project with organic farmers in Germany [68,69]. Because of the comparatively low dispersal ability of *C. rosae*, organic farmers in Germany are advised to move their carrot fields to a distance of 1 km away from fields of the previous year, when substantial *C. rosae* infestations have been detected.

Another approach is to harvest the carrots before the larvae of the second generation of *C. rosae* attack the main root (i.e., the marketable product). In Sweden, the development of damage by *C. rosae* is predicted using a degree-day model which gives information on when to harvest in order to avoid damage to the marketable part of the plant [70]. This harvesting model has been incorporated into the UK forecasting system for carrot fly and is being tested in Norway at the moment.

### 4.4. Biological Control

For crops where the marketable part of the plant is affected by the pest (i.e., those with a low damage threshold), methods of control that are less ‘efficient’ than insecticides or crop covers may be impractical, unless two or more approaches are integrated to increase the overall level of control. However, for leafy brassicas in particular, control methods that reduce, rather than eliminate, pest infestations may be viable. Such crops are most susceptible when they are very young, before their root systems have developed.

All the root feeding fly species are affected by generalist predators and by parasitoids (such as *Trybliographa rapae* Hymenoptera: Cynipoidae and *Aleochara bilineata* Coleoptera: Staphylinidae). Parasitoids attack the pest either at the larval or pupal stage, but, in both cases, the pest does not die until after some level of damage has been inflicted on the crop. Thus, parasitoids cannot be deployed to provide timely control, but the larval parasitoid *T. rapae* and the pupal parasitoid *A. bilineata* are often common in brassicaceous crops and impose relatively high parasitisation levels in *D. radicum* [71,72], thereby contributing to the regulation of the pest population from generation to generation.

In contrast, the impact of predators affects prey populations immediately. Finch et al. [73] investigated the release of artificially reared *A. bilineata* as a predator of *D. radicum* eggs. They discounted the feasibility of using it as a predator of eggs, mainly because its effectiveness at finding eggs had been overestimated previously [73]. It was also an ineffective predator of first-instar larvae. However, Finch et al. [73] did envisage a role as a predator of second instar-larvae and, as part of this, they foresaw a role for decision support in terms of indicating the optimum timing(s) for the augmentative release of the predators. There were a number of other problems associated with releasing these beetles, a key one being the inability to predict ahead what the size of the infestation would be, and, therefore, the number of beetles that should be released (since each has a fixed capacity to consume larvae). As *D. radicum* is such a mobile insect, it is very hard to predict the size of the infestation, although it is possible to monitor adult numbers or egg-laying in real time (which might be too late to respond). Due to the various limitations of the approach, this research was not taken further. A similar approach was pursued subsequently in the UK with another predatory staphylinid beetle, *Atheta coriaria,* that might be reared by biocontrol companies. It was envisaged that the beetles might be applied to modules during propagation or after planting out [74]. Again, advance information on the phenology and (if feasible) the abundance of *D. radicum* would be useful. To date, this approach has not been taken further. In Germany, experiments in the field and in climate chambers were conducted with regard to releasing the predatory mite *Macrocheles robustulus* (Berlese) to reduce the numbers of eggs of *D. radicum*. The predatory mite is produced commercially and offered for biological control in glasshouse crops. Initial results showed that there is potential for reducing the numbers of eggs of *D. radicum* [7,75]. Predators may, therefore, mostly have impact on fly pest populations through habitat management practices in conservation biological control strategies [76,77], although such approaches may be compromised by intra-guild predation and competition [78,79].

Techniques that could have more traction practically are the deployment of microbial biocontrol agents, such as entomopathogenic fungi or nematodes [3,4,5,6,80]. Both types of organisms are commercially available for biological control in several European countries, but their efficacy is challenged by the difficulty of identifying the best application method for reaching the fly larvae; by the need for large dosages to impose sufficient mortality in the larval population; and by the difficulty of consistent control under field conditions. Methods for application of the fungus *Metarhizium brunneum* and conditioning of entomopathogenic nematodes for local soil temperatures have been investigated in the current FlyIPM project in the context of deployment of the biocontrol agents below ground to leafy brassicas for control of *D. radicum*. Drenches of fungal conidia have been shown to provide control of *D. radicum* [5], but this application method is impractical for the grower. As the target is the larvae of *D. radicum, M. brunneum* was applied as a granular product to the planting modules before transplanting to the field so that the fungus is established as a preventative measure when egg laying commences. Whilst information on the size of the infestation would not be so critical, information on the likely periods of peak egg-laying would be useful. However, transplanting of modules in the spring may be at a time when temperatures are too low for the fungi to be effective. Studies have shown that the parasitoid *T. rapae* is able to locate high densities of prey (i.e., *D. radicum* larvae), but also avoid host habitats with high densities of the entomopathogenic fungus *M. brunneum* [81]. A combination of habitat management for parasitoids and inoculation with entomopathogenic fungi may be an effective integrated approach against *D. radicum* larvae, although there may be no additive effect [82].

### 4.5. Manipulation of Pest Behaviour

For *D. radicum* in particular, methods of manipulating pest behaviour to reduce egg-laying on susceptible crops have been investigated using semiochemicals and/or by growing more than one plant species in the cropped area. The latter has included approaches using undersowing, companion planting, intercropping [83,84], volatiles decreasing plant infestation and increasing egg predation [17,85], and trap crops [14,86,87].

**Figure 2:**
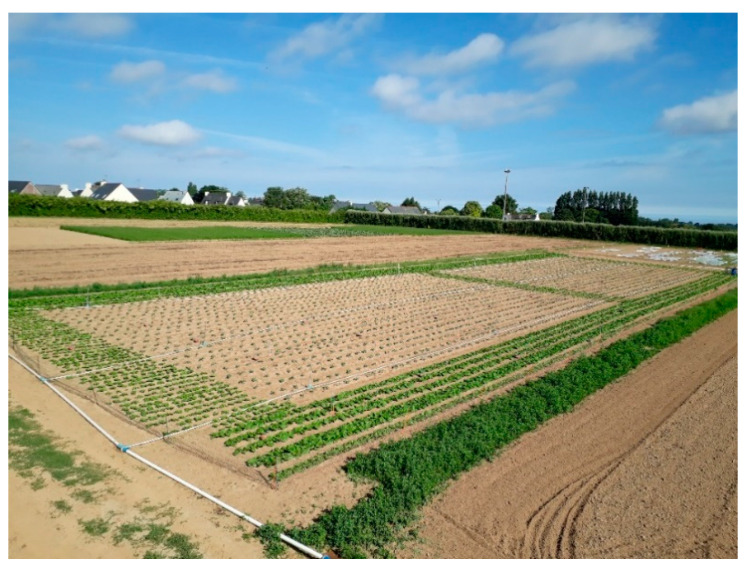
Field trial to evaluate a ‘push–pull strategy’ to manage *Delia radicum* in broccoli using Chinese cabbage (outer rows) as a trap crop [87] (image provided by Fabrice Lamy and Anne-Marie Cortesero, University of Rennes, France).

The most sophisticated utilisation of behavioural manipulation in agriculture is summarised under the term ‘push–pull strategy’. It describes the use of deterrent stimuli to keep cash crops free from pests, while attractive stimuli are used to concentrate them in areas where control is facilitated. The most popular example makes use of many components: push plants fix nitrogen, increase soil fertility and decrease the need for fertilizer, and deterrence of the pest is accompanied by control of a root-parasitic weed; pull plants simultaneously attract pests and parasitoids, inhibit pest species development and are used as fodder for livestock [88]. Altogether, the strategy is deployed in small farming systems. In industrial cultivation of cabbage, onions and carrots, such multifunctional components are lacking and the substitution of a certain amount of cash crop for trap plants tends to be economically adverse unless decreased field size outweighs the losses to pest infestations. Push plants generally compete with the cash crop for nutrients and often lack explicit deterrent effects; therefore, intercropping is rather used for support of natural enemies and the push effect is better accomplished by timed application of chemical deterrents. Since the beginning of research in this area, vegetable root flies have been a target for the ’push–pull strategy’: Miller and Cowles [89] who were among the first to conceptualize the push–pull method, recommended the combined use of a deterrent (cinnamaldehyde) and an alternative oviposition site (onion culls) to protect young seedlings from *D. antiqua*. They specifically inferred from their experiments that this method would reliably diminish field losses to 3–5% in onion fields with infestation of 10,000 to 30,000 pupae/ha in Iowa, USA. For push–pull control of root flies in brassicaceous crops, Chinese cabbage has proved to be a very attractive plant for the cabbage root fly, concentrating a large part of the infestation pressure (Figure 2), but it is also a perfectly suitable plant for the development of the fly and cannot be used as a dead-end trap crop [87]. Therefore, additional measures to prevent thriving pest populations from increasing risks at the next generation need to be taken. These can include the use of physical measures, such as mechanical destruction (see, for example, Deguine et al. [90]) or targeted augmentative biological control. An optimum efficacy of the latter will again depend on the right time for taking action.

For all these approaches, knowledge of the phenology of the target pest species, and the development rates of the plant species used, would be important to ensure that all plant species, crops and ‘companions’, are at the appropriate stage of growth to achieve the optimal effect, and that semiochemicals are applied at the right time. Missing the main peak of egg laying is likely to mitigate the repellent effect of semiochemicals dramatically.

## 5. Climate Change

Weather conditions in Europe are altering as a result of climate change, and Europe has also warmed faster than any other continent in recent decades [91]. Since the 1980s, warming has been greatest at high latitudes, particularly in the winter [92]. For example, in Norway, the mean temperature for the years 2009–2018 has been 1.1 °C warmer than the 1961–1990 mean [93]. All but one of the years from 1997 to 2019 has had mean temperatures above the 1961–1990 mean. The exception was 2010, which was one of the coldest years since 1900 (1.0 °C colder than normal). The highest mean temperature in recent years was recorded in 2014, 2.2 °C above average [93]. In the UK, the situation is about the same, and the mean temperature over the decade 2009–2018 has been 0.9 °C warmer than the 1961–1990 mean. Additionally, the 10 warmest years for the UK (since 1884), have occurred since 2002 [94].

Climate change will affect the geographic distribution and phenology of pest insect species. Phenological and other types of models can be very useful in the development of scenarios. For example, Ning et al. [95] considered the current potential and future world-wide distribution of *D. antiqua* using maximum entropy ecological niche modelling. The impact of climate change on the phenology of *D. radicum* was considered thirty years ago using the forecasting model developed in the UK [96]. This suggested that, as mean temperatures increased, emergence of flies in the spring would be earlier and less synchronised, as the completion of diapause and post-diapause development would occur at the same time in different individuals within the population. It was also suggested that aestivation by pupae when the weather was particularly warm would disrupt egg-laying. More recently, the phenology of *C. rosae* over recent years in Switzerland has been described [97]. There were three complete generations in years with wet summers (2007, 2014). However, the flight of the ‘third’ generation after summers with pronounced hot periods was extremely weak at many locations (in 2006, 2013, 2015 and 2017). This is likely to be due to a delay in the second generation, as the soil temperatures at 10 cm depth in June or July were over 23 °C for several weeks, which is likely to have increased the mortality of larvae and pupae and also led to a temporary pause in pupal development due to aestivation. Similar effects have been observed in France [98], where the ‘second’ generation does not occur in very hot years.

In the context of climate change, the use of decision support tools coupled with monitoring activities can provide information about changes in phenology that arise as an adaptation to increasing temperatures or other factors (e.g., earlier emergence, additional generations). Whilst it is over a longer time-scale, this information gives an indication of what the future structure of crop production and associated crop protection might be, adding value to the information that decision support tools, and the experts that manage them, can provide. This information is likely to be of use at several points in the supply chain.

## 6. Benefits Provided by the Use of Decision Support Tools

**Figure 3:**
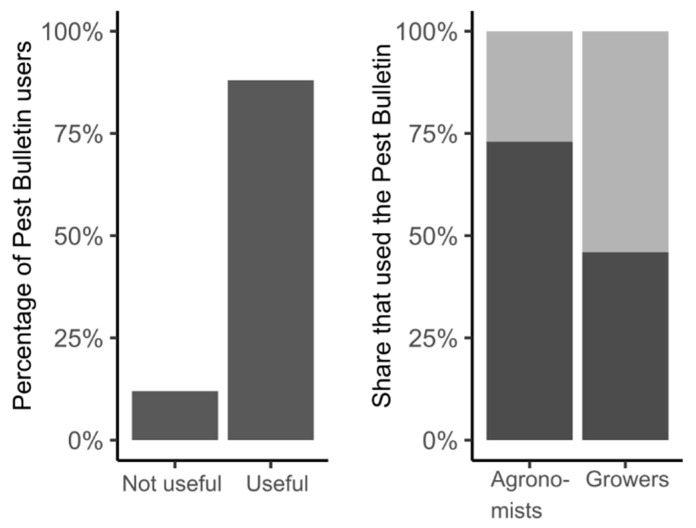
Summary of the results of a questionnaire survey conducted by the Agriculture and Horticulture Development Board (AHDB) to establish the broad range of uses of pest monitoring and forecasting information disseminated to vegetable growers in Britain through the AHDB Pest Bulletin [100].

There is a lack of published evidence of the benefits provided by the use of decision support tools in terms of reduced and/or better-targeted interventions for the management of the pest insects considered in this review. These benefits would ideally be expressed in some relevant currency such as financial savings or environmental risk reduction. Studies have been undertaken for some other pests of field crops and, for example, Ferguson et al. [99] go some way towards this in the context of the management of *Meligethes* spp. on oilseed rape, making a comparison between simple rule-based advice and a phenological-model-based decision support system. For the species considered in this review, a questionnaire survey conducted by the Agriculture and Horticulture Development Board (AHDB) established the broad range of uses of pest monitoring and forecasting information disseminated to vegetable growers in Britain through the AHDB Pest Bulletin (including information on *D. radicum*, *D*. *platura*, *D. florilega* and *C. rosae*) and an estimate of ‘use’ and ‘value’ [100]. The vast majority (88%) of Pest Bulletin users rated it as either very useful or quite useful, and use was higher amongst agronomists than growers (73% versus 46%) (Figure 3). Of the options provided in the questionnaire, users indicated that targeting in-field monitoring was the most common way the Pest Bulletin supported decision making, followed by targeting of insecticide sprays and determining spray frequency. Selection of plant protection products was the least common response. Other uses for the information included: supporting their own field observations, comparing numbers to previous years, collecting data for personal interest, and to keep informed/ahead of emerging issues. Just 12% of users felt that the Pest Bulletin did not support their decision making. This questionnaire was disseminated primarily to ensure that there was sufficient interest in the AHDB Pest Bulletin for the AHDB to continue to fund it and it in no way tried to assess the ‘true’ value of the information provided. Going forward, an evidence-based estimation of the economic and environmental paybacks could promote the acceptance, and thus greater use, of decision support tools by growers, as well as supporting the development of additional phenological models for established and newly-occurring pest species.

For the pests considered in this review, there is also an opportunity to develop more sophisticated decision support systems for pest management, integrating phenological models with geographical information systems to track infestations and population changes and also to include support for recommendations for interventions. A number of such systems are reviewed by Damos [20]. However, this would require considerable investment in research and development, for crops which provide a relatively small market for such technologies compared with cereal crops, for example. There is also the question of the sustainability of such systems, as, for example, both of the more complex simulation models described here [47,48] were developed as part of research projects and are now in urgent need of software upgrades.

## 7. Conclusions

At present, there is a change in the paradigm within Europe with regard to crop protection and, in the future, it will be necessary to rely increasingly on an integrated approach using tools which separately have less immediate efficacy than pesticides. In simple terms, there will be fewer ‘silver bullets’. Knowledge of insect phenology is promoted as a key element of IPM strategies, and the aim of this article is to consider the approaches taken to monitor and forecast the occurrence of root-feeding fly pests of vegetables, how they are used currently in pest management, and how they might be used in the future. It has highlighted some approaches where such information is very useful for decision support, for example, the management of *C. rosae* with insecticidal sprays and the management of all the pests considered using crop covers. There are other approaches, particularly those that need to be applied at sowing or transplanting, where knowledge of phenology and abundance is less necessary. Going forward, it is likely that the number of insecticidal control options available in Europe will diminish. Some of the alternative methods of control will be less ‘effective’ and/or persistent and will need to be applied in combination, such as the application of repellents and trap crop management. For these, access to information about pest phenology and abundance is likely to be very important.

## Figures and Tables

**Table 1:** Main root-feeding fly pests of vegetable crops and the types of decision-support tools available in Western Europe (see text for further details).

Pest Insect	Common Name	Plant Family Affected	Monitoring Systems	Forecasting Systems	Thresholds Available
*Delia radicum*	Cabbage root fly	Brassicaceae	Traps using vision and olfaction, egg sampling	Degree-day models, simulation models	Norway, France
*Delia floralis*	Turnip fly	Brassicaceae	Traps using vision, egg sampling	Warnings are disseminated based on egg counts which are related to damage thresholds	Norway
*Delia platura*	Bean seed fly	Various	Traps using vision and olfaction	Degree-day models	No
*Delia florilega*	Root fly	Various	Traps using vision and olfaction	No	No
*Delia antiqua*	Onion fly	Alliaceae	Traps using vision	Degree-day models, simulation model	No
*Chamaepsila rosae*	Carrot fly	Asteraceae	Traps using vision	Degree-day models, simulation models	Several countries
